# Perioperative neutrophil:lymphocyte ratio and postoperative NSAID use as predictors of survival after lung cancer surgery: a retrospective study

**DOI:** 10.1002/cam4.428

**Published:** 2015-03-10

**Authors:** Jae Eun Choi, John Villarreal, Javier Lasala, Vijaya Gottumukkala, Reza J Mehran, David Rice, Jun Yu, Lei Feng, Juan P Cata

**Affiliations:** 1 University of California, San Diego School of MedicineCalifornia; 2 Department of Anesthesiology and Perioperative Medicine, The University of Texas MD Anderson Cancer CenterHouston, Texas; 3 Department of Thoracic and Cardiovascular Surgery, The University of Texas MD Anderson Cancer CenterHouston, Texas; 4 Department of Biostatistics, The University of Texas MD Anderson Cancer CenterHouston, Texas; 5 Outcomes Research ConsortiumCleveland, Ohio

**Keywords:** Anti-inflammatory agents, nonsteroidal, neoplasms, non–small-cell lung cell, surgery, inflammation

## Abstract

The association between neutrophil:lymphocyte ratio (NLR) and poor long-term outcomes in patients with non–small-cell lung cancer (NSCLC) has been demonstrated in numerous studies. The benefit of perioperative administration of anti-inflammatory drugs on these outcomes has not been well established. Our aim in this retrospective study was to investigate the effects of postoperative nonsteroidal anti-inflammatory drug (NSAID) administration and NLR on tumor recurrence and survival in patients' undergoing surgical resection for NSCLC. This retrospective study included perioperative data from 1139 patients who underwent surgical resection for stages I–III NSCLC. Perioperative data such as baseline characteristics, adjuvant or neoadjuvant therapy, pre- and postoperative NLR, and NSAID use (ketorolac, ibuprofen, celecoxib, or in combination) were included. We evaluated the association between preoperative NLR and NSAID use on recurrence-free (RFS) and overall survival (OS). In all, 563 patients received an NSAID as a part of their postoperative management. The majority of patients received ketorolac (*n* = 374, 67.16%). Ketorolac administration was marginally associated with better OS (*P* = 0.05) but not with RFS (*P* = 0.38). Multivariate analysis (*n* = 1139) showed that preoperative NLR >5 was associated with a reduction in RFS (hazard ratio [HR] = 1.37; 95% confidence interval [CI] = 1.05–1.78; *P* = 0.02) and OS (HR = 1.69; 95% CI = 1.27–2.23; *P* = 0.0003). However, after accounting for tumor stage, NLR ≥5 was a predictor of RFS and OS only in patients with stage I NSCLC. To conclude, preoperative NLR was demonstrated to be an independent predictor of RFS and OS in a subset of patients with early stage NSCLC. Ketorolac administration was not found to be an independent predictor of survival.

## Introduction

Lung cancer is still the leading cause of death among women and men in the United States 1. Surgery remains the mainstay treatment option for patients with non–small-cell lung cancer (NSCLC) 2; therefore, a number of perioperative-related factors including tumor stage, margin status, type of resection, and type of anesthetic and analgesic technique have been investigated with the goal of predicting and improving long-term survival 3,4.

Neutrophils are key cellular components of the inflammatory system and lymphocytes play an important role in immune surveillance and immune editing. Therefore, the neutrophil:lymphocyte ratio (NLR) correlates those two cellular components as a marker of perioperative inflammation 5,6. In the context of cancer, an NLR >5 has been suggested as an independent prognostic factor for decreased recurrence-free survival (RFS) in patients with malignancies, which highlights the importance of perioperative inflammation and immune suppression in oncological outcomes 7–10.

It has been speculated that the use of nonsteroidal anti-inflammatory drugs (NSAIDs) during and after surgery could not only modify the tumor microenvironment in which micrometastasis are present but also reduce migration and invasion of circulating malignant cells; therefore, the administration of these drugs in the perioperative period might have a significant impact on cancer recurrence 11–13. The perioperative administration of NSAIDs (ketorolac or diclofenac) has been shown to be an independent risk factor for distant metastasis-free survival, and ketorolac administration, specifically, was a predictor of better overall survival (OS) in an observational study of patients with stage I or II NSCLC 8.

The aim of this retrospective study was to determine possible associations between preoperative NLR and postoperative use of NSAIDs on RFS and OS in patients with stage I–III NSCLC undergoing curative resection. We hypothesized that patients with a low inflammatory status (NLR <5) and taking NSAIDs postoperatively have a longer RFS and OS.

## Methods

Study approval and waiver of written informed consent was obtained from the University of Texas MD Anderson Cancer Centre Institutional Review Board (IRB) prior to the start of the study.

Perioperative data were collected, stored, and managed in a REDCap (Research Electronic Data Capture) database from patients who underwent surgical resection for primary stage I, II, or III NSCLC between January 2004 and December 2010 at The University of Texas MD Anderson Cancer Center Centre was included in this retrospective analysis. Patients 18 years or older who had surgery with the intention to cure were included in the analysis. Those who had palliative surgery or secondary malignancies were excluded from the analysis. The analyzed data included patient age, gender, body mass index (BMI), American Society of Anesthesiology (ASA) physical status, tumor histology, WHO tumor stage (I, II, or III), type of surgery, and administration of neoadjuvant and/or adjuvant chemotherapy and/or radiation. Intraoperative anesthetic care of the patients consisted of general balanced anesthesia typically involving the use of a volatile anesthetic in oxygen, intravenous opioids, muscle relaxation with a nondepolarizing agents, and normothermia.

Postoperative management typically consisted of patient-controlled epidural analgesia with or without the addition of nonsteroidal anti-inflammatory drugs that started within 72 h after surgery. Patients who were administered an NSAID were given ketorolac (30–60 mg/day), ibuprofen (200–800 mg/day), or celecoxib (200–300 mg/day) alone or in combination. The pre- and postoperative NLR was calculated from laboratory results obtained within 2 weeks before surgery and on postoperative day 1. The NLR of subsequent postoperative days was not calculated because routine blood specimens were not collected beyond postoperative day 1 in all the patients included in the analysis.

### Statistical analysis and endpoints

The primary end points were RFS and OS (OS). RFS was defined as the time in months from the date of surgery to either the date of first evidence of recurrence or the date of death, whichever happened first. OS was defined as the time in months from the date of surgery to the date of death. Patients who were lost to follow up, remained disease-free, or were alive at the time of analysis were censored at the last known follow-up date.

Descriptive statistics for demographics and baseline patient characteristics included mean and standard deviation (SD) for continuous variables, while frequency counts and percentages were used for categorical variables. Fisher's exact test or chi-square test was used to evaluate the association between two categorical variables. Kruskal–Wallis test was used to evaluate the difference in a continuous variable between patient groups. Wilcoxon signed-rank test was used to compare the pre- and postoperative change in continuous variables. A cut-off value of 5 was used to discriminate between patients with high and low preoperative NLR (NLR ≥5 or <5) 8. RFS and OS were analyzed using the Kaplan–Meier method and log-rank tests were used to assess the difference in time-to-event outcomes between patient groups. The median time-to-event in months and survival rates at 3 and 5 years were calculated including 95% confidence intervals. Univariate Cox proportional hazard models were used to evaluate differences in OS and RFS for continuous variables. Multivariable Cox proportional hazard models were used for multivariate analysis to include covariates that have shown to be associated with prognosis in previous studies or showed a *P* < 0.2 in the univariate analysis. *P* < 0.05 were considered statistically significant. Statistical software SAS 9.1.3 (SAS, Cary, NC) and S-Plus 8.0 (TIBCO Software Inc., Palo Alto, CA) were used for all the analyses.

## Results

### Patient characteristics

Data from 1139 patients were included in this retrospective analysis. The mean age of our patient sample was 64.73 years (SD = 10.42) and included 602 males (52.85%). Approximately half of the patients were diagnosed as stage I (*n* = 606; 53.20%). Six hundred and thirty (55.31%) patients were determined to have an adenocarcinoma and 509 (44.69%) had a different histological type of NSCLC. Most of the patients had a thoracotomy (*n* = 905; 79.46%) and did not receive neoadjuvant chemotherapy (*n* = 894; 78.49%) or radiation therapy (*n* = 1121; 98.42%), or adjuvant chemotherapy (*n* = 854; 74.98%) or radiation therapy (*n* = 969; 85.07%) (Table 1). Tumor stage was found to be significantly associated with age (*P* = 0.002), gender (*P* = 0.0008), tumor histology (*P* = 0.0001), neoadjuvant chemotherapy (*P* < 0.0001) and radiation therapy (*P* = 0.0001), adjuvant chemotherapy (*P* < 0.0001) and radiation (*P* < 0.0001) or surgery type (*P* < 0.0001).

**Table 1:** Characteristics of 1139 patients who underwent resection for stage I, II, or III primary NSCLC (2004–2010)

Variable	
Age, mean (SD)	64.73 (10.42)
BMI, mean (SD)	27.1 (5.35)
Gender, *n* (%)	
Male/female	602 (52.85)/537 (47.15)
ASA physical status, *n* (%)	
1	3 (0.26)
2	121 (10.62)
3	963 (84.55)
4	52 (4.57)
WHO tumor stage, *n* (%)	
I	606 (53.20)
II	253 (22.21)
III	280 (24.58)
Adenocarcinoma histology, *n* (%)	
Yes/no	630 (55.31)/509 (44.69)
Type of surgery, *n* (%)	
Thoracotomy/thoracoscopy	905 (79.46)/234 (20.54)
Chemotherapy, *n* (%)	
Preoperative (yes/no)	245 (21.51)/894 (78.49)
Postoperative (yes/no)	285 (25.02)/854 (74.98)
Radiation, *n* (%)	
Preoperative (yes/no)	18 (1.58)/1121 (98.42)
Postoperative (yes/no)	170 (14.93)/969 (85.07)
NLR, mean (SD)	
Preoperative/postoperative	2.95 (2.43)/10.28 (6.74)
Postoperative NSAID use (any), *n* (%)	
Yes/no	563 (49.43)/576 (50.57)
Ketorolac only, *n* (%)	
Yes/no	374 (32.84)/765 (67.16)
Ibuprofen only, *n* (%)	
Yes/no	232 (20.37)/907 (79.63)
Celecoxib only, *n* (%)	
Yes/no	46 (4.04)/1093 (95.96)

NSCLC, non–small-cell lung cancer; SD, standard deviation; BMI, body mass index; ASA, American Society of Anesthesiology; WHO, World Health Organization; NLR, neutrophil:lymphocyte ratio; NSAID, nonsteroidal anti-inflammatory drug.

The average preoperative NLR was 2.95 (SD = 2.43) and increased postoperatively to 10.28 (SD = 6.74) (*P* < 0.0001). There was a significant difference in preoperative NLR values and tumor stage with stage II having the highest NLR (mean = 3.25, SD = 2.34), followed by stage III (mean = 3.11, SD = 3.37) and stage I (mean = 2.75, SD = 1.86). One hundred and ten (9.66%) patients had a preoperative NLR ≥5. There was a trend toward a statistically significant difference between preoperative NLR group and tumor stage so that the patients with a high NLR (≥5) had higher tumor stage (stage I = 7.8% vs. stage II = 11.9% and stage III = 11.8%) (*P* = 0.07).

In regard to the perioperative use of NSAIDs, 563 (49.4%) patients were administered an NSAID. Three hundred and seventy-four of them received ketorolac alone or in combination with ibuprofen and/or celecoxib (Table 1). There were no statistically significant differences between the use of NSAIDs among patients with different tumor stages (*P* = 0.436). The average preoperative NLR of patients treated with NSAIDs (mean = 2.89, SD = 2.11) was not statistically different to that of patients who did not received NSAIDs (mean = 3.01, SD = 2.7, *P* = 0.259). Also, the proportion of patients with a preoperative NLR higher than 5 who were treated with NSAIDs (*n* = 55, 9.8%) or not (*n* = 55, 9.5%) was nearly identical (*P* = 0.899). Remarkably, the NSAIDs use were associated with a decrease in the rate of patients with a NLR higher than 5. Briefly, 79.4% (*n* = 447) of the patients with a NLR higher than 5 belonged to NSAIDs group compared to 84.7% (*n* = 488, *P* = 0.191). Although the average postoperative NLR was lower in patients who received NSAIDs (mean = 9.98, SD = 6.64) compared to those who did not receive the treatment (mean = 10.58, SD = 6.82), the difference was not statistically significant (*P* = 0.060).

### Recurrence-free survival

The median RFS of the overall general population was 71.16 months (95% confidence interval [CI] = 65.44–80.32) and RFS rates at 3 and 5 years were 0.64 (95% CI = 0.61–0.67) and 0.55 (95% CI = 0.52–0.58), respectively. Univariate analysis demonstrated that a higher preoperative NLR (≥5) was predictive of poor RFS (*P* = 0.0003), whereas a postoperative NLR was not (*P* = 0.51). Additionally, older age, male, ASA 3–4, higher tumor stage, neoadjuvant chemotherapy, adjuvant radiation, and thoracotomy were associated with decreased RFS (Table 2). In multivariate analysis, a preoperative NLR >5 (hazard ratio [HR] = 1.37; 95% CI = 1.05–1.78; *P* = 0.02), older age (HR = 1.03; 95% CI = 1.02–1.04; *P* < 0.0001), higher tumor stage (II vs. I: HR = 1.64, 95% CI = 1.32–2.04, *P* < 0.0001; III vs. I: HR = 2.07, 95% CI = 1.63–2.61, *P* < 0.0001), neoadjuvant chemotherapy (HR = 1.41; 95% CI = 1.15–1.74; *P* = 0.001), and adjuvant radiation (HR = 1.47; 95% CI = 1.1.6–1.86; *P* = 0.002) were associated with lower RFS (Table 3).

**Table 2:** Univariate analysis for RFS and OS

Variable	*N*	Event	RFS HR (95% CI)	Median RFS time in months (95% CI)	RFS rate at 5 years (95% CI)	*P*-value	Event	OS HR (95% CI)	Median OS time in months (95% CI)	OS rate at 5 years (95% CI)	*P*-value
Age (continuous)	1139	547	1.02 (1.01–1.03)			<0.0001	432	1.03 (1.02–1.04)			<0.0001
BMI (continuous)	1139	547	0.99 (0.98–1.01)			0.331	432	1.00 (0.98–1.02)			0.845
Gender											
Female	537	235		81.7 (71.09–NA)	0.6 (0.56–0.64)	0.0025	178		NA (102.14–NA)	0.71 (0.67–0.75)	0.0005
Male	602	312		57.75 (50.56–72.67)	0.5 (0.46–0.54)		254		95.24 (76.38–NA)	0.61 (0.57–0.65)	
ASA											
1–2	124	51		96.85 (63.24–68.79)	0.62 (0.53–0.72)	0.088	37		108.28 (98.49–NA)	0.74 (0.67–0.83)	0.0527
3–4	1015	496		XX (62.29–77.6)	0.54 (0.5–0.57)		395		102.89 (86.86–NA)	0.65 (0.62–0.68)	
Stage											
1	606	223		99.77 (87.19–NA)	0.67 (0.63–0.71)	<0.0001	165		NA (114.72–NA)	0.77 (0.74–0.81)	<0.0001
2	253	137		52.4 (42.54–72.7)	0.47 (0.41–0.54)		111		85.51 (65.97–NA)	0.59 (0.53–0.66)	
3	280	187		21.55 (17.25–30.85)	0.35 (0.3–0.42)		156		52.04 (44.38–74.64)	0.47 (0.41–0.53)	
Adenocarcinoma											
No	509	235		76.38 (64.85–NA)	0.55 (0.51–0.6)	0.635	190		114.72 (94.74–NA)	0.65 (0.61–0.7)	0.9899
Yes	630	312		68.4 (60.35–78.52)	0.54 (0.5–0.58)		242		97.86 (85.51–NA)	0.66 (0.62–0.7)	
Neoadjuvant chemo											
No	894	392		82.19 (71.78–97.86)	0.59 (0.56–0.63)	<0.0001	313		109.66 (97.86–NA)	0.7 (0.66–0.73)	<0.0001
Yes	245	155		28.48 (19.51–44.42)	0.38 (0.32–0.45)		119		62.94 (51.41–NA)	0.51 (0.44–0.58)	
Adjuvant chemo											
No	854	402		71.75 (64.98–83.51)	0.55 (0.52–0.59)	0.6722	319		102.89 (95.24–NA)	0.66 (0.62–0.69)	0.996
Yes	285	145		71.09 (53.25–81.8)	0.53 (0.47–0.6)		113		98.49 (81.8–NA)	0.66 (0.6–0.72)	
Adjuvant radiation											
No	969	425		82.92 (71.75–97.86)	0.59 (0.55–0.62)	<0.0001	333		114.72 (99.77–NA)	0.7 (0.67–0.73)	<0.0001
Yes	170	122		22.21 (17.25–33.64)	0.32 (0.25–0.4)		99		50.85 (45.34–71.02)	0.42 (0.35–0.51)	
Thoracotomy											
No	234	81		95.24 (76.38–NA)	0.66 (0.6–0.73)	<0.0001	60		NA (95.24–NA)	0.76 (0.71–0.83)	0.0005
Yes	905	466		65.9 (55.22–72.7)	0.52 (0.48–0.55)		372		97.86 (83.38–NA)	0.63 (0.6–0.66)	
Preoperative											
NLR ≥5	110	67		31.11 (19.51–57.92)	0.39 (0.3–0.5)	0.0003	60		55.22 (39.29–NA)	0.46 (0.37–0.57)	<0.0001
NLR <5	1029	480		72.7 (67.54–83.51)	0.56 (0.53–0.6)		372		108.28 (95.93–NA)	0.68 (0.65–0.71)	
Postoperative											
NLR ≥5	935	454		71.02 (62.94–77.6)	0.54 (0.51–0.58)	0.509	355		108.28 (91.49–NA)	0.65 (0.62–0.69)	0.978
NLR <5	204	93		81.7 (63.24–NA)	0.57 (0.5–0.65)		77		102.14 (82.62–NA)	0.67 (0.61–0.75)	
NSAIDs (any)											
No	576	285		68.4 (57.26–83.51)	0.53 (0.49–0.57)	0.663	232		99.77 (82.92–NA)	0.63 (0.59–0.67)	0.178
Yes	563	262		72.7 (65.44–86.86)	0.56 (0.52–0.61)		200		102.89 (94.74–NA)	0.68 (0.64–0.73)	
Ketorolac											
No	765	373		69.97 (58.44–80.32)	0.53 (0.5–0.57)	0.383	301		97.86 (82.92–NA)	0.63 (0.59–0.67)	0.054
Yes	374	174		75.33 (63.24–98.49)	0.57 (0.52–0.63)		131		109.66 (95.93–NA)	0.71 (0.66–0.76)	
Celecoxib											
No	1093	524		71.35 (65.54–80.32)	0.55 (0.52–0.58)	0.3434	412		102.89 (95.2–NA)	0.66 (0.63–0.69)	0.1869
Yes	46	23		51.08 (20.27–NA)	0.49 (0.36–0.67)		20		NA (51.08–NA)	0.55 (0.41–0.73)	
Ibuprofen											
No	907	445		68.79 (62.94–81.8)	0.54 (0.51–0.58)	0.862	356		102.89 (94.74–NA)	0.65 (0.62–0.69)	0.774
Yes	232	102		72.7 (68.07–NA)	0.58 (0.51–0.65)		76		NA (72.7–NA)	0.67 (0.61–0.74)	

RFS, recurrence-free survival; HR, hazard ratio; CI, confidence interval; OS, overall survival; BMI, body mass index; Chemo, chemotherapy; ASA, American Society of Anesthesiology; NLR, neutrophil:lymphocyte ratio; NSAID, nonsteroidal anti-inflammatory drug.

**Table 3:** Multivariate Cox proportional hazard model for RFS in all 1139 patients who underwent resection for stage I, II, and III NSCLC (2004–2010)

Covariate	HR (95% CI)	*P*-value
Age	1.029 (1.02–1.04)	<0.0001
ASA		
2 versus 1	0.5 (0.12–2.06)	0.3377
3 versus 1	0.51 (0.13–2.07)	0.3497
4 versus 1	0.91 (0.22–3.8)	0.8971
Stage		
II versus I	1.638 (1.317–2.037)	<0.0001
III versus I	2.064 (1.633–2.609)	<0.0001
Neoadjuvant chemotherapy: yes versus no	1.414 (1.15–1.74)	0.001
Adjuvant radiation: yes versus no	1.469 (1.16–1.86)	0.0015
Thoracoscopy versus thoracotomy	0.796 (0.62–1.02)	0.071
Preoperative NLR: high (≥5) versus low (<5)	1.367 (1.05–1.78)	0.019
Postoperative NLR: high (≥5) versus low (<5)	0.971 (0.77–1.22)	0.803

RFS, recurrence-free survival; NSCLC, non–small-cell lung cancer; HR, hazard ratio; CI, confidence interval; ASA, American Society of Anesthesiology; NLR, neutrophil:lymphocyte ratio.

**Figure 1:**
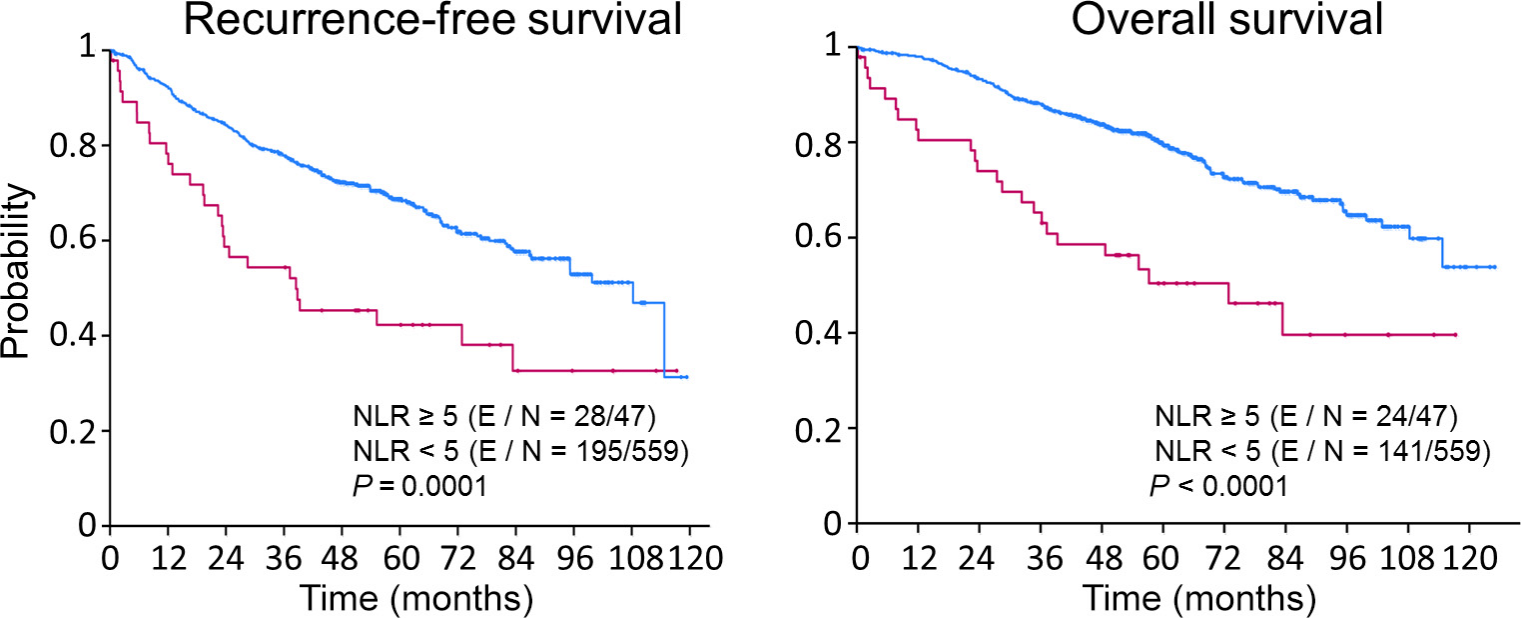
Kaplan–Meier curves of recurrence-free survival (*n* = 606) and overall survival (*n* = 606) for patients who underwent resection for stage I primary non–small-cell lung cancer (NSCLC) (2004–2010).

The effect of preoperative NLR on RFS in patients with stage I NSCLC was statistically significant in the univariate Cox proportion analysis (*P* < 0.0001) but was not for patients with stage II (*P* = 0.51) or stage III (*P* = 0.56) NSCLC (Fig. 1). After adjustment for age, ASA 3–4, NSAIDs use, neoadjuvant chemotherapy, adjuvant radiation, thoracoscopy, and postoperative NLR in the multivariate Cox proportional hazard model, the analysis demonstrated that a preoperative NLR ≥5 was an independent predictor of poor RFS patients in patients with stage I disease (HR = 2.13, 95% CI = 1.42–3.19, *P* = 0.0002) (Table 4).

**Table 4:** Multivariate Cox proportional hazard model for RFS in 606 patients who underwent resection for stage I NSCLC (2004–2010)

Covariate	HR (95% CI)	*P*-value
Age	1.037 (1.022–1.053)	<0.0001
ASA 3/4 versus 1/2	1.40 (0.860–2.279)	0.18
NSAID versus no NSAID	0.931 (0.708–1.225)	0.61
Neoadjuvant chemotherapy: yes versus no	1.479 (0.973–2.249)	0.07
Adjuvant radiation: yes versus no	1.981 (1.069–3.671)	0.03
Thoracoscopy versus thoracotomy	0.881 (0.643–1.206)	0.43
Preoperative NLR: high (≥5) versus low (<5)	2.132 (1.424–3.193)	0.0002
Postoperative NLR: high (≥5) versus low (<5)	0.820 (0.575–1.170)	0.27

RFS, recurrence-free survival; NSCLC, non–small-cell lung cancer; HR, hazard ratio; CI, confidence interval; ASA, American Society of Anesthesiology; NSAID, nonsteroidal anti-inflammatory drug; NLR, neutrophil:lymphocyte ratio.

For the overall population of patients (*n* = 1139), postoperative NSAID administration was not predictive of RFS (any NSAID: *P* = 0.66, ketorolac: *P* = 0.48, celecoxib: *P* = 0.34, ibuprofen *P* = 0.86). When data were analyzed by tumor stage, postoperative use of NSAIDs was not associated with longer RFS (stage I, *P* = 0.12; stage II, *P* = 0.35 and stage III, *P* = 0.19). The postoperative NLR was not a predictor of RFS in patients who did (*P* = 0.526) or did not (*P* = 0.826) receive NSAIDs.

### Overall survival

The median OS for the entire population of patients was 102 months (95% CI = 94.74–NA). OS rates at 3 and 5 years were 0.76 (95% CI = 0.74–0.79) and 0.66 (95% CI = 0.63–0.69), respectively. The predictors of decreased RFS, including high preoperative NLR (≥5), older age, male, ASA 3–4, higher tumor stage, neoadjuvant chemotherapy, adjuvant radiation, and thoracotomy, were also the predictors of decreased OS (Table 2). A preoperative NLR ≥5 (HR = 1.69; 95% CI = 1.27–2.23; *P* = 0.0003), age (HR = 1.04; 95% CI = 1.03–1.05), postoperative radiation (HR = 1.48; 95% CI = 1.15–1.92), and higher tumor stage (II vs. I: HR = 1.72, 95% CI = 1.34–2.21, *P* < 0.0001; III vs. I: HR = 2.34, 95% CI = 1.80–3.04, *P* < 0.0001) were also associated with shorter OS in the multivariate analysis. A postoperative NLR ≥5 was not predictive of poor OS (HR = 0.84; 95% CI = 0.65–1.08; *P* = 0.18).

Univariate analysis demonstrated that only patients with stage I cancer who had a preoperative NLR ≥5 showed a worse OS survival than those with a lower than 5 NLR (*P* < 0.0001) (Fig. 1). The multivariate Cox proportional hazard model showed that the effect of a preoperative NLR ≥5 on OS was significant after adjusting for age, gender, ASA 3–4, NSAIDs use, adjuvant chemotherapy and radiation, thoracoscopy, and postoperative NLR (HR = 2.72; 95% CI = 1.75–4.23; *P* < 0.0001) (Table 5). Overall, NSAID, celecoxib or ibuprofen, administration was not predictive of OS (NSAID: *P* = 0.18, celecoxib *P* = 0.19, ibuprofen *P* = 0.77) when all stages were included in the analysis. However, patients who were administered ketorolac demonstrated a marginally better OS (*P* = 0.05) in univariate analysis. When the analysis was conducted by tumor stage, univariate analysis demonstrated longer OS only for stage I patients who received NSAIDs compared with those not treated with these analgesics (*P* = 0.03) (Fig. 1). This association was not significant when adjusting for covariates including age, gender, ASA 3–4, adjuvant chemotherapy and radiation, type of surgery, and pre- and postoperative NLR (HR = 0.91; 95% CI = 0.65–1.26; *P* = 0.58) (Table 6). The data analysis also demonstrated that the postoperative NLR was not a predictor of OS in patients who did (*P* = 0.64) or did not (*P* = 0.706) receive NSAIDs.

**Table 5:** Multivariate Cox proportional hazard model for OS in all 1139 patients who underwent resection for stage I, II, and III NSCLC (2004–2010)

Covariate	HR (95% CI)	*P*-value
Age	1.037 (1.03–1.05)	<0.0001
ASA		
2 versus 1	0.357 (0.09–1.50)	0.159
3 versus 1	0.391 (0.1–1.58)	0.1879
4 versus 1	0.768 (0.18–3.23)	0.7187
Stage		
II versus I	1.722 (1.34–2.2)	<0.0001
III versus I	2.339 (1.8–3.04)	<0.0001
Neoadjuvant chemotherapy: yes versus no	1.20 (0.95–1.512)	0.1358
Adjuvant radiation: yes versus no	1.483 (1.15–1.922)	0.0029
Thoracoscopy versus thoracotomy	0.787 (0.590–1.050)	0.1032
Preoperative NLR: high (≥5) versus low (<5)	1.686 (1.274–2.230)	0.0003
Postoperative NLR: high (≥5) versus low (<5)	0.841 (0.654–1.083)	0.1796

OS, overall survival; NSCLC, non–small-cell lung cancer; HR, hazard ratio; CI, confidence interval; ASA, American Society of Anesthesiology; NLR, neutrophil:lymphocyte ratio.

**Table 6:** Multivariate Cox proportional hazard model for OS in 606 patients who underwent resection for stage I NSCLC (2004–2010)

Covariate	HR (95% CI)	*P*-value
Age	1.051 (1.032–1.071)	<0.0001
Gender: male versus female	1.593 (1.154–2.199)	0.005
ASA: 3/4 versus 1/2	1.692 (0.886–3.232)	0.11
NSAID versus no NSAID	0.913 (0.657–1.268)	0.59
Neoadjuvant chemotherapy: yes versus no	1.334 (0.819–2.173)	0.25
Adjuvant radiation: yes versus no	1.766 (0.858–3.634)	0.12
Thoracoscopy versus thoracotomy	0.780 (0.534–1.140)	0.20
Preoperative NLR: high (≥5) versus low (<5)	2.724 (1.753–4.233)	<0.0001
Postoperative NLR: high (≥5) versus low (<5)	0.805 (0.533–1.217)	0.30

OS, overall survival; NSCLC, non–small-cell lung cancer; HR, hazard ratio; CI, confidence interval; ASA, American Society of Anesthesiology; NSAID, nonsteroidal anti-inflammatory drug; NLR, neutrophil:lymphocyte ratio.

## Discussion

This study demonstrates that a high preoperative NLR (≥5), a marker of inflammation, is associated with a decrease in RFS and OS in patients with early stage NSCLC. These results are in agreement with those reported by Forget et al. and Sarraf et al. who in a much smaller population of patients with stage I and II NSCLC demonstrated that a preoperative NLR ≥5 was an independent risk factor for worse RFS and OS 3,14. Others have also shown similar results; however, in those studies the cut-off NLR was 2.5, which makes difficult the comparison with our results 15,16.

Tumor stage might influence perioperative inflammation and immune suppression; therefore, we further analyzed the data to determine whether an NLR ≥5 was also a predictor of survival when considering tumor stage separately. A high preoperative NLR (≥ 5) was shown to be a significant independent predictor of decreased RFS and OS independent in patients with stage I disease but not stage II or III. Consistent with the findings reported by Sarraf et al., we found that the mean NLR was slightly, but statistically significantly higher in patients with tumor stage II and III than those in stage I, indicating a higher inflammatory status and perhaps more immune suppression in patients with more advanced stages of NSCLC 14. This suggests, at least in our patient population, that preoperative immune suppression may be more important in patients with limited tumor burden. In addition, patients with higher stages are more likely to have longer, complex, and more invasive surgeries which are perioperative factors associated with a greater stress and inflammatory response 17,18. Therefore, it could be argued that our results may reflect the association between type of surgery, early recovery, and improved survival; although this is merely speculative because there was only a trend toward NLR ≥5 in patients with stage III disease (*P* = 0.084).

NSAIDs are commonly given intra- and postoperatively as adjuvant analgesics. It has been suggested that the perioperative use of NSAIDs could be associated with prolonged RFS and OS 19,20. Several mechanisms have been proposed including modulation of the inflammatory response and a direct positive effect on the tumor microenvironment and invasiveness of cancer cells 12. Forget et al. demonstrated that intraoperative NSAID use was an independent predictor of increased distant metastasis-free survival, especially when given at the beginning of surgery, but not locoregional recurrence in patients with breast cancer 20. Additionally, the study found that intraoperative ketorolac administration was an independent predictor of increased OS. In our work, we found that NSAID administration within 72 h after surgery was not predictive of longer OS. However, postoperative ketorolac administration (alone or in combination with other NSAIDS) showed a trend toward a significant association with an increase in OS but not RFS. These findings support previous studies suggesting that the addition of ketorolac, specifically, may have a positive prognostic effect on long-term survival 20,21.

Our study has several limitations, therefore, the results should be interpreted with caution. First, this is a retrospective study; hence there are unknown factors that might have affected the studied outcomes. Second, the impact of immune suppression, inflammation, and effect of NSAIDs could be confounded by the histological type of tumor, type of recurrence (locoregional recurrence vs. distant metastasis), and adjuvant treatment. Moreover, the total amount of NSAIDs administered postoperatively was not quantified and this could potentially explain the discordant findings between the study by Forget et al. and our work. Third, the statistical power was limited by the number of patients available in our database and therefore there is a possibility of a type II error. A propensity score based on logistic regression was not conducted because the number of covariates included in the logistic regression model is generally calculated as the number of patients in the smallest group divided by 10 22. Therefore, with few patients per group by stage, we could put only four covariates in the model when in fact there were several covariates. Finally, the generalizability of our findings might be limited because our patient population was extracted from a large tertiary care only dedicated to cancer care.

In conclusion, a high preoperative NLR (≥5) was predictive of longer RFS and OS following resection in patients with stage I NSCLC. NSAID administration was not found to be an independent predictor of survival, however, postoperative ketorolac administration showed a trend toward better OS. Despite the relatively small number of patients receiving NSAIDs and the retrospective nature of this study, the results of this study provide support for future prospective studies comparing the effect of tumor stage, perioperative inflammatory status, and perioperative NSAIDs.
